# Supplementary material for: The Protective Effect of (-)-Tetrahydroalstonine against OGD/R-Induced Neuronal Injury via Autophagy Regulation
Source: Molecules. 2023 Mar 4;28(5):2370. doi: 10.3390/molecules28052370 (PMC10005631; doi:10.3390/molecules28052370)
Supplement: Supplementary file 1 [file molecules-28-02370-s001.zip › molecules-2171219-supplementary.pdf]

## Supplementary Materials

# The Protective Effect of (-)-Tetrahydroalstonine against OGD/R-Induced Neuronal Injury via Autophagy Regulation

Yumei Liao [1,2,†], Jun-Ya Wang [3,†], Yan Pan [2,†], Xueyi Zou [2,3,†], Chaoqun Wang [2], Yinghui Peng [2], Yun-Lin Ao [3], Mei Fong Lam [4], Xiaoshen Zhang[1], Xiao-Qi Zhang [3,5,*], Lei Shi [1,2], Shiqing Zhang [2,*]

[1] *Department of Cardiovascular Surgery, The First Affiliated Hospital, Jinan University, Guangzhou 510632, China*

[2] *JNU-HKUST Joint Laboratory for Neuroscience and Innovative Drug Research, College of Pharmacy, Jinan University, Guangzhou 510632, China*

[3] *Guangdong Provincial Engineering Research Center for Modernization of TCM, College of Pharmacy, Jinan University, Guangzhou 510632, China*

[4] *Centro Hospitalar Conde de São Januário, Macau, China*

[5] *NMPA Key Laboratory for Quality Evaluation of TCM, Jinan University, Guangzhou 510632, China*

† These authors contributed equally to the study.

* Corresponding author: sqzhang@jnu.edu.cn (Shiqing Zhang); xqzhang74@hotmail.com (Xiao-Qi Zhang)

### *Identification of (–)-Tetrahydroalstonine*

**(–)-Tetrahydroalstonine**: $[\alpha]_D^{25}$ -93.5 (c 0.90, $CH_3OH$); UV ($CH_3OH$) $\lambda_{max}$ (log ε) 226 (3.92), 285 (0.76); IR (KBr) $\nu_{max}$ 3420, 1698, 2875, 1627, 1541, 1455 $cm^{-1}$; HR-ESI-MS at *m/z* 353.1789 $[M+H]^+$ (calcd for $C_{21}H_{25}N_2O_3$, 353.1787); $^1$H-NMR ($CD_3OD$, $\delta_H$ = 4.87 ppm, 600 MHz), $\delta_H$: 7.55 (1H, s, H-17), 7.35 (1H, d, *J* = 7.8, H-9), 7.26 (1H, d, *J* = 7.8, H-12), 7.00 (1H, t, *J* = 7.8, H-11), 6.93 (1H, t, *J* = 7.8, H-10), 4.48 (1H, m, H-19), 3.73 (3H, s, $COOCH_3$), 3.32 (1H, m, H-3), 3.15 (1H, d, *J* = 12.5, H-21a), 3.00 (1H, m, H-5a), 2.92 (1H, m, H-6a), 2.74 (1H, m, H-15), 2.71-2.64 (3H, overlapped, H-21b, H-6b and H-14a), 2.51 (1H, m, H-5b), 1.69 (1H, m, H-20), 1.42 (1H, m, H-14b), 1.37 (3H, d, *J* = 6.2,H-18); $^{13}$C-NMR ($CD_3OD$, $\delta_C$ = 49.0 ppm, 150 MHz), $\delta_C$: 169.6 (s, C-22), 156.9 (s, C-17), 138.1 (s, C-13), 135.6 (s, C-2), 128.3 (s, C-8), 121.8 (s, C-11), 119.7 (s, C-10), 118.5 (s, C-9), 112.0 (s, C-12), 110.9 (s, C-16), 108.0 (s, C-7), 73.6 (s, C-19), 61.8 (s, C-3), 57.1 (s, C-21), 54.8 (s, C-5), 51.7 (s, C-23),39.9 (s, C-20), 34.8 (s, C-14), 32.7 (s, C-15), 22.5 (s, C-6), 18.9 (s, C-18). The parameters of the spectrograms are shown in the follwing figures.

## Supplementary Figures

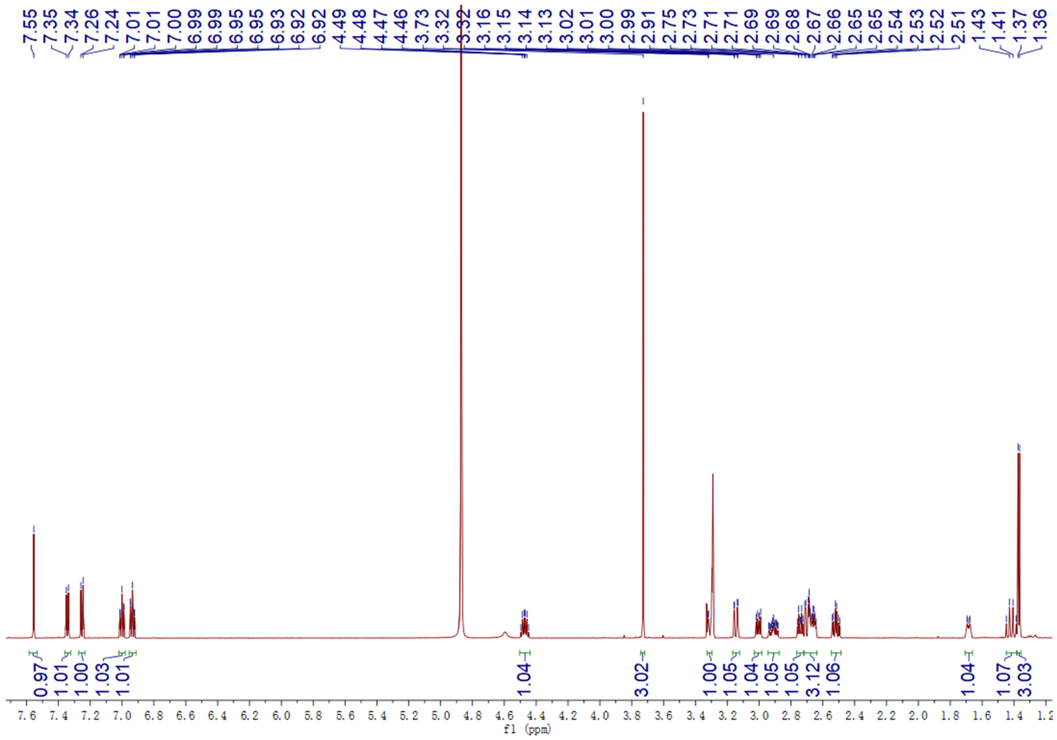


**Figure S1.** $^1$H NMR spectrum of (–)-Tetrahydroalstonine (600 Hz, $CD_3OD$)

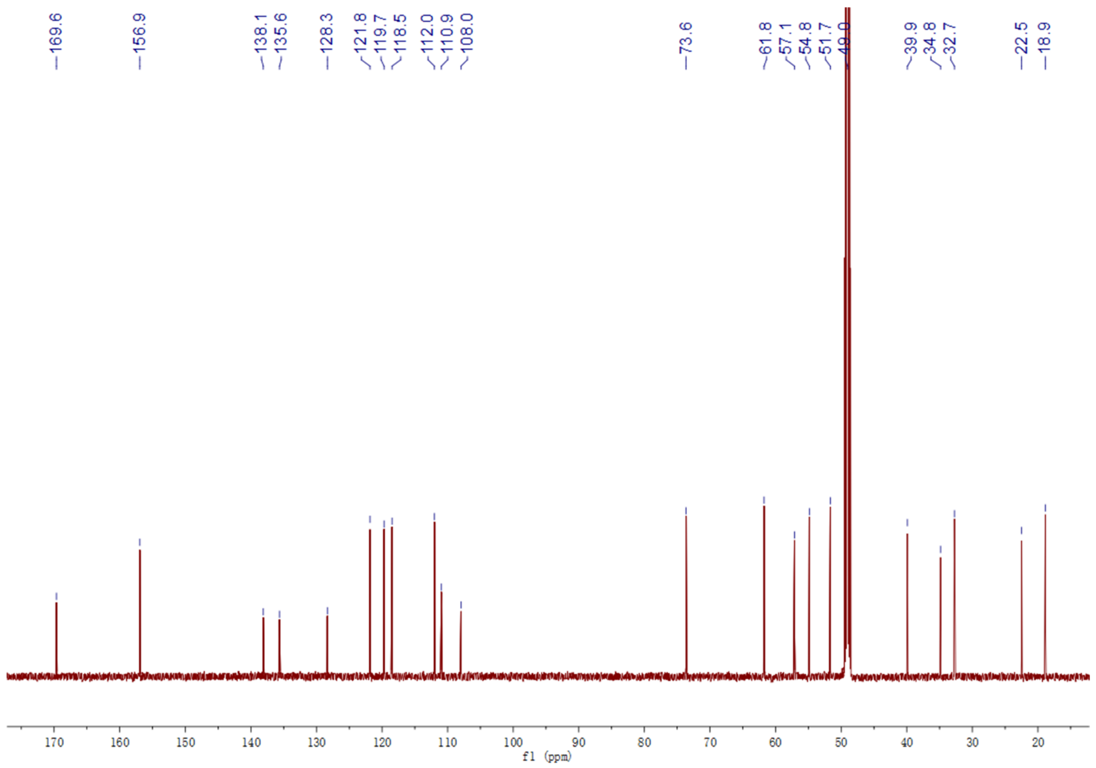


**Figure S2.** $^{13}C$ NMR spectrum of (–)-Tetrahydroalstonine (150 Hz, $CD_3OD$)

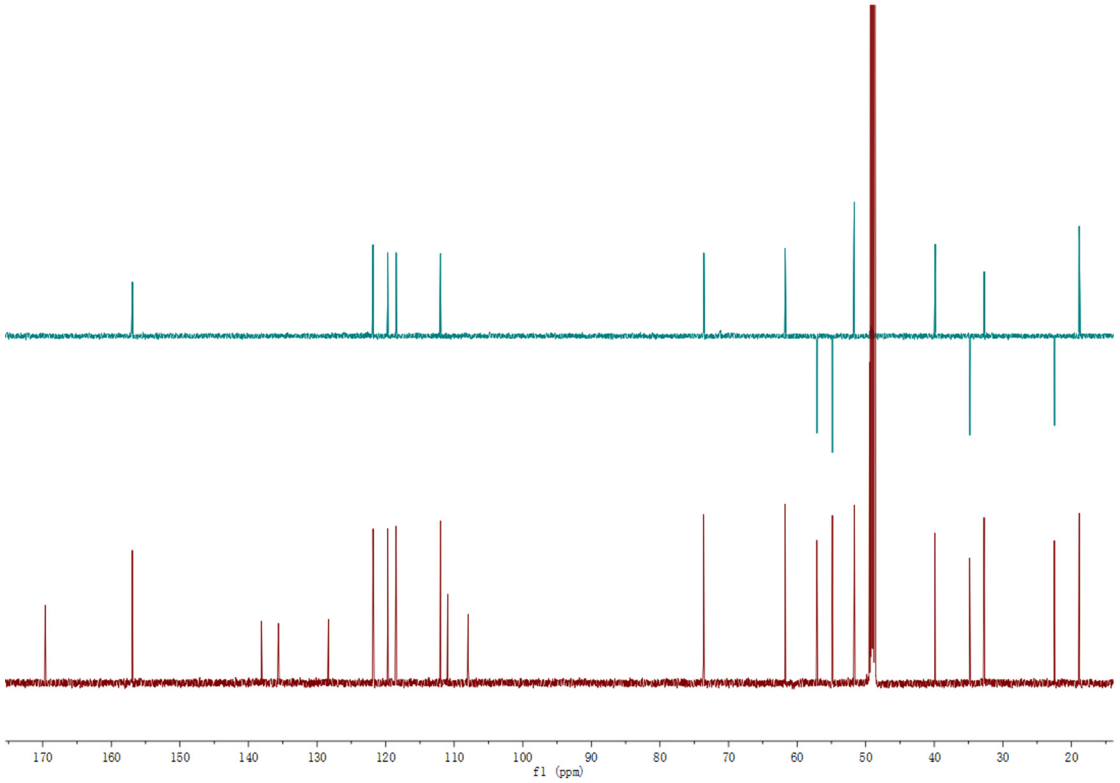


**Figure S3.** DEPT-135 spectrum of (–)-Tetrahydroalstonine (150 Hz, $CD_3OD$)
